# PROGNOSTIC ACKNOWLEDGEMENT IN OLDER ADULT CANCER PATIENTS

**DOI:** 10.1093/geroni/igad104.3016

**Published:** 2023-12-21

**Authors:** Sophia Maggiore, Mary Kate Koch, Carma Bylund, Susan Bluck

**Affiliations:** University of Florida, Gainesville, Florida, United States; University of Florida, Gainesville, Florida, United States; University of Florida, Gainesville, Florida, United States; University of Florida, Gainesville, Florida, United States

## Abstract

Even with improved prognostic transparency (Chochinov et al., 2000) individuals struggle to acknowledge a serious-terminal prognosis. Patients who acknowledge their condition, however, show less distress and increased end-of-life planning (Ray et al., 2006). Aims were to: (1) examine extent older adults with serious cancer acknowledge their illness, and (2) identify factors predicting acknowledgement. Individuals receiving outpatient palliative care (N=204; Mage=65.78 years; SDage=7.43) completed measures of Symptom Severity, the Patient Dignity Inventory (DPI; Chochinov et al., 2008), and Illness Acknowledgement (i.e., healthy, seriously ill, terminally ill). Participants with Stage 4 cancer were more likely to acknowledge serious or terminal illness than Stage 1-3 patients, X2 (2,181)=12.93, p <.01), though only 31% of Stage 4 patients reported being terminally ill. In addition, ANOVA showed Symptom Severity, F(2, 190) = 9.86, p < .001 differed by level of acknowledgement. Those rating themselves as relatively healthy had less severe symptoms than seriously or terminally ill groups. To further predict acknowledgement, DPI subscales were examined. Dependency predicted acknowledgement of serious-terminal illness (Odds Ratio = .75, p <.05). A final model, controlling for age and education, showed Symptom Severity (OR = 1.03, p <.01) and DPI Dependency (OR = .79, p <.05) predicted acknowledging serious-terminal illness versus lack of acknowledgement. Though often relied on in communicating to patients, cancer stage alone may not help older adults with cancer to acknowledge their prognosis. By identifying influences leading to illness acknowledgement, future research can examine positive and negative outcomes during end-of-life coping.

